# Increase of avian *Plasmodium circumflexum* prevalence, but not of other malaria parasites and related haemosporidians in northern Europe during the past 40 years

**DOI:** 10.1186/s12936-022-04116-7

**Published:** 2022-03-24

**Authors:** Gediminas Valkiūnas, Mélanie Duc, Tatjana A. Iezhova

**Affiliations:** Nature Research Centre, Akademijos 2, 08412 Vilnius, Lithuania

**Keywords:** *Plasmodium*, Birds, Transmission, Haemosporidian parasites, Vector-born infections, Invasive malaria

## Abstract

**Background:**

Malaria is a health problem not only in human and veterinary medicine, but also in wildlife. Several theoretical studies have suggested that avian malaria transmission might be increasing in Europe. However, there are few direct empirical observations. Research on the distribution of avian haemosporidian parasites was initiated around the Curonian Lagoon, Europe in 1976 and continues since. This has provided an opportunity to compare the prevalence and diversity of avian malaria parasites (genus *Plasmodium*) and related haemosporidians (genera *Haemoproteus* and *Leucocytozoon*) in the same bird species using similar methodology but examined in two groups 40 years apart. This study aimed to describe and discuss the available data on this subject.

**Methods:**

Prevalence and diversity of haemosporidians was compared in two passeriform bird groups, which consisted of the same species that were sampled on the coast of the Curonian Lagoon (Russia, Lithuania) during the same season (September) in 1978–1983 (bird Group 1) and 2020 (bird Group 2). Blood films of the European robin*,* Coal tit*,* Great tit*,* Eurasian wren*,* and Eurasian jay were screened by microscopic examination. Parasites were identified using morphological characters of blood stages. PCR-based methods were applied to determine genetic lineages of the parasites found in birds of Group 2.

**Results:**

No difference was discernible in the prevalence or diversity of haemosporidian parasites belonging to *Haemoproteus, Leucocytozoon, Plasmodium* (*Haemamoeba*) and *Plasmodium* (*Novyella*) between birds of Groups 1 and 2. This indicates a similar rate of transmission and relatively stable epidemiological situation in regard of these infections during the past 40 years. The prevalence of only one malaria parasite species, *Plasmodium* (*Giovannolaia*) *circumflexum,* increased remarkably, but only in Coal tit*,* Great tit*,* and Eurasian wren, with no significant prevalence change in European robin and Eurasian jay.

**Conclusion:**

*Plasmodium circumflexum* is spreading and seems to be a new invasive avian malaria pathogen in countries with cold climates. The exceptionally high prevalence of *P. circumflexum* in birds breeding in relatively close-nests suggests an important role of the nesting biology related to bird-vector interaction in this pathogen transmission. The epidemiological situation seems to be relatively stable in regard of other studied avian hosts and haemosporidian parasites in northern Europe.

## Background

Malaria is a common disease of reptiles, birds and mammals. It is caused by numerous species of haemosporidian parasites belonging to the genus *Plasmodium*. The biggest malaria parasite diversity has been described in countries with warm climates where these pathogens flourish in various terrestrial vertebrates [1–3]. In countries with temperate climates, *Plasmodium* species are relatively rare in reptiles and mammals [4, 5], but are prevalent and diverse in many bird populations [6, 7]. Malaria is a health problem not only in human and veterinary medicine, but also in wildlife [1, 8–10]. This disease can be severe in non-adapted avian hosts and is an alarming bird health problem globally [11–14].

Numerous recent studies have suggested that avian malaria transmission might expand in regions with cold climates in association with global warming [15, 16]. However, this conclusion might be biased due to the application of relatively complicated research methodology, in which the data on bird infection with malaria parasites were gathered from different, often unrelated publications, summarized and finally applied in mathematical models. Such approach should be regarded as hypothetical [17, 18]; it may be useful by providing theoretical information about possible trends occurring in nature, but remains insufficiently convincing in regard of ecology and life cycles of *Plasmodium* parasites and other haemosporidians. The life cycles and persistence of these parasites remain poorly known or completely unknown at the species level [19]. This is a particularly sensitive issue if the primary data for mathematical modelling came from different studies, which were based on blood samples collected from different regions and seasons, different bird species and ages as well as the application of different microscopic examination protocols with different sensitivity. Each of these factors might significantly influence the prevalence data [7, 19–21] and complicate understanding of even major trends in the epidemiological situation. Furthermore, the available mathematical models on malaria parasite distribution have usually been designed for application at the level of genus *Plasmodium* and did not consider the diversity of these pathogens at the subgenus or species level. Avian *Plasmodium* parasites are diverse, with over 50 species described and classified in five subgenera, whose members have different life cycles and modes of development in their avian hosts, and often use different vector species for transmission [19, 22–24]. More straightforward monitoring research based on certain bird populations and parasite species would be helpful for better understanding of changes in avian malaria epidemiology. This issue is worth better understanding due to the climate warming, which is known to affect some bird populations and might significantly influence pathogen distribution by migrating birds in the future [25–30]. Such empirical data on this subject that are available [31–34] often agrees with theoretical predictions [17, 35]. However, these observations were limited in timescale and remain at the level of hypothesis in regard of a long-time perspective.

Research on the distribution and prevalence of avian malaria parasites and other haemosporidians was initiated around the Curonian Lagoon, Europe in 1976 [36, 37], and continues to this day. A big dataset has been collected, providing opportunities to compare prevalence of avian malaria parasites (genus *Plasmodium*) and related haemosporidians (genera *Haemoproteus* and *Leucocytozoon*) in common European bird species during the past 40-years using uniform methodology. This study aimed to compare the prevalence and diversity of haemosporidian parasites in selected bird species, sampled in the same area in the same season, but 40 years apart. A remarkable increase in prevalence of *Plasmodium (Giovannolaia) circumflexum* infection was found, but not for any other malaria parasite or *Haemoproteus* and *Leucocytozoon* species.

## Methods

**Fig. 1:**
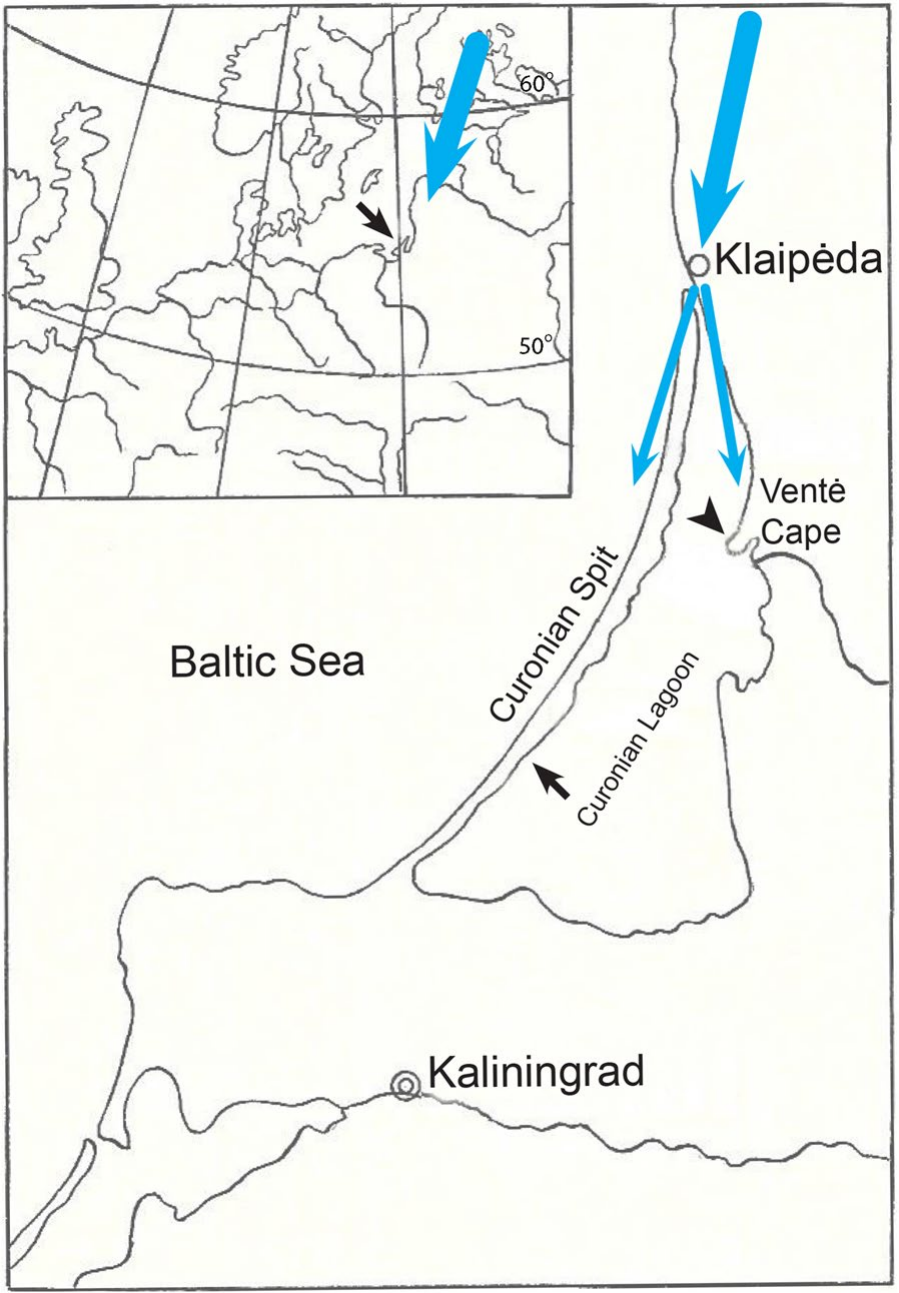
General scheme of location of the study sites on the Curonian Spit (short black arrow) and Ventė Cape (arrowhead). Blue thick arrow shows the main migration direction of examined birds from breeding areas to wintering grounds. When the migrating birds reach the Klaipėda region during autumnal migration, the migratory flow divides, and the birds of same populations continue their migration along the coast of the Curonian Lagoon via the Curonian Spit and Ventė Cape where they were sampled. Further explanations were given in the text

Two groups of passeriform birds were caught at two close sites located on the coast of the Curonian Lagoon, Europe (Fig. 1, Table 1). The birds of Group 1 were sampled at the Curonian spit (55°5ʹ2ʹʹ N, 20°44′6ʹʹ E, Biological station of Zoological institute, Russian Academy of Sciences) in September 1979–1983. The birds of Group 2 were sampled at Ventė Cape (55°20′27ʹʹ N, 21°11′22ʹʹ, Ventės ragas Ornithological station, Lithuania) in September 2020. The material was collected during the birds’ autumnal migration towards wintering grounds. All sampled birds were migrating along the so-called White Sea–Baltic Sea migration route [37, 38]. Ringing data indicate that the majority of them originated in the regions of northeast Europe located between the White See and the Baltic Sea (Karelia, Finland, Leningrad oblast of the Russian Federation, Baltic states and neighbouring regions) ([38–40], www.vros.lt, Ventės Ragas Ornithological Station database, accessed in December 2021). Although the precise sites of their hatching and infection with haemosporidian parasites are unknown, all juveniles certainly originated in regions located north of the sampling sites. All examined birds prefer to migrate over land. When they reach the Klaipeda region, the migration route splits in two streams: some individuals fly over the Curonian Spit, and some move along the southern coast of the Curonian Lagoon and reach Ventė Cape (Fig. 1, blue arrows). Ringing data show that the birds caught at both study sites belong to the same populations ([38, 39], www.vros.lt, Ventės Ragas Ornithological Station database).

**Table 1:** Prevalence of avian malaria parasites (*Plasmodium*) and related haemosporidians in passeriform birds migrating to wintering grounds from their breeding sites located between the White Sea and the Baltic Sea, northern Europe.

Bird family, bird species and parasites	Bird group, study site and period of sampling				Difference, *p*-value ^a^
	Group 1, Curonian Spit, September 1979–1983		Group 2, Ventes Ragas peninsular, September 2020		
	No. examined	No. infected ^b^	No. examined	No. infected ^b^	
Muscicapidae					
*Erithacus rubecula* ^c^	33	4 (12.1)	40	3 (7.5)	0.04 (0.8350)
Infected with:					
*Haemoproteus* sp.		1 (3.0)		1 (2.5)	0.34 (0.5582)
*Plasmodium* sp.		1 (3.0)		1 (2.5)	0.34 (0.5582)
*Leucocytozoon* sp.		2 (6.1)		1 (2.5)	0.02 (0.8887)
Paridae					
*Periparus ater* ^d^	60	3 (5.0)	40	25 (62.5)	**19.33 (0.0000)**
Infected with:					
*Haemoproteus* sp.		3 (5.0)		7 (17.5)	2,23 (0. 1354)
*Plasmodium* sp.		0		18 (45.0)	**19.64 (0.0000)**
*Leucocytozoon* sp.		0		0	0 ^e^
*Parus major* ^c^	39	13 (33.3)	40	27 (67.5)	2.42 (0.1195)
Infected with:					
*Haemoproteus* sp.		7 (17.9)		10 (25.0)	0.12 (0. 7296)
*Plasmodium* sp.		2 (5.1)		15 (37.5)	**6.62 (0.0101)**
*Leucocytozoon* sp.		4 (10.2)		2 (5.0)	0.15 (0.6939)
Troglodytidae					
*Troglodytes troglodytes* ^d^	17	0	40	17 (42.5)	**5.00 (0.0253)**
Infected with:					
*Haemoproteus* sp.		0		0	0 ^e^
*Plasmodium* sp.		0		17 (42.5)	**5.00 (0.0253)**
*Leucocytozoon* sp.		0		0	0 ^e^
Corvidae					
*Garrulus glandarius* ^d^	30	12 (40.0)	29	16 (55.2)	0.22 (0.6404)
Infected with:					
*Haemoproteus* sp.		2 (6.7)		6 (20.7)	0.99 (0.3191)
*Plasmodium* sp.		0		4 (13.8)	2.11 (0.1462)
*Leucocytozoon* sp.		11 (36.7)		6 (20.7)	0.54 (0.4630)
Total infected with					
*Haemoproteus* sp.		13 (7.3)		24 (12.7)	1.95 (0.1628)
*Plasmodium* sp.		3 (1.7)		55 (29.1)	**36.99 (0.0000)**
*Leucocytozoon* sp.		17 (9.5)		9 (4.8)	2,09 (0.1478)
Grant total	179	33 (18.4)	189	83 (46.6)	**16.07 (0.0001)**

The birds were sampled on the coast of the Curonian Lagoon at two close study sites (Curonian Spit and Ventė Cape, see Fig. 1). Only data of microscopic examination of blood films was given

^a^Yates-corrected Chi-squire test value, followed in parenthesis by the corresponding significance *P*-value. Significant differences were shown in bold

^b^Number of infected birds, followed in parenthesis by prevalence of infection (in percentage)

^c^Only juvenile birds were samples

^d^Bird age was not identified. During autumnal migration, the juvenile birds predominate in all bird species, and likely predominated during this study, but the presence of some adult individuals cannot be ruled out in both sampled periods

^e^Parasites were not reported in both groups

Blood samples were examined by the same researchers (GV and TAI) using the same protocol of microscopic examination [19]. Blood films were prepared on ready-to-use glass slides, fixed with methanol and stained with Giemsa. Approximately 200,000 red blood cells were screened under × 900 or × 1000 magnification to determine parasite positive individuals. Intensity of parasitaemia was estimated in percentage by counting the number of parasites observed per 10,000 red blood cells. The statistical analysis was carried out using the ‘Statistica 7ʹ package. Prevalences of infection were compared by Yates corrected Chi-square (χ^2^) test. A *p*-value of ≤ 0.05 was considered significant.

Bird species and age were identified by professional ornithologists. Five common European bird species (European robin *Erithacus rubecula,* Coal tit *Periparus ater,* Great tit *Parus major,* Eurasian wren *Troglodytes troglodytes,* Eurasian jay *Garrulus glandarius*) were examined, with 179 and 189 individuals tested in the Group 1 and the Group 2, respectively (Table 1). In both groups, birds of the same species and age were present. Only juvenile (hatch year) individuals of European robin and Great tit were used in this study. They can be readily distinguished from adults due to plumage coloration [41]. While the age of Coal tits, Eurasian wrens and Eurasian jays was not determined, juvenile birds are known to constitute the predominant part in samples of each of these species during autumnal migration [42].

**Fig. 2:**
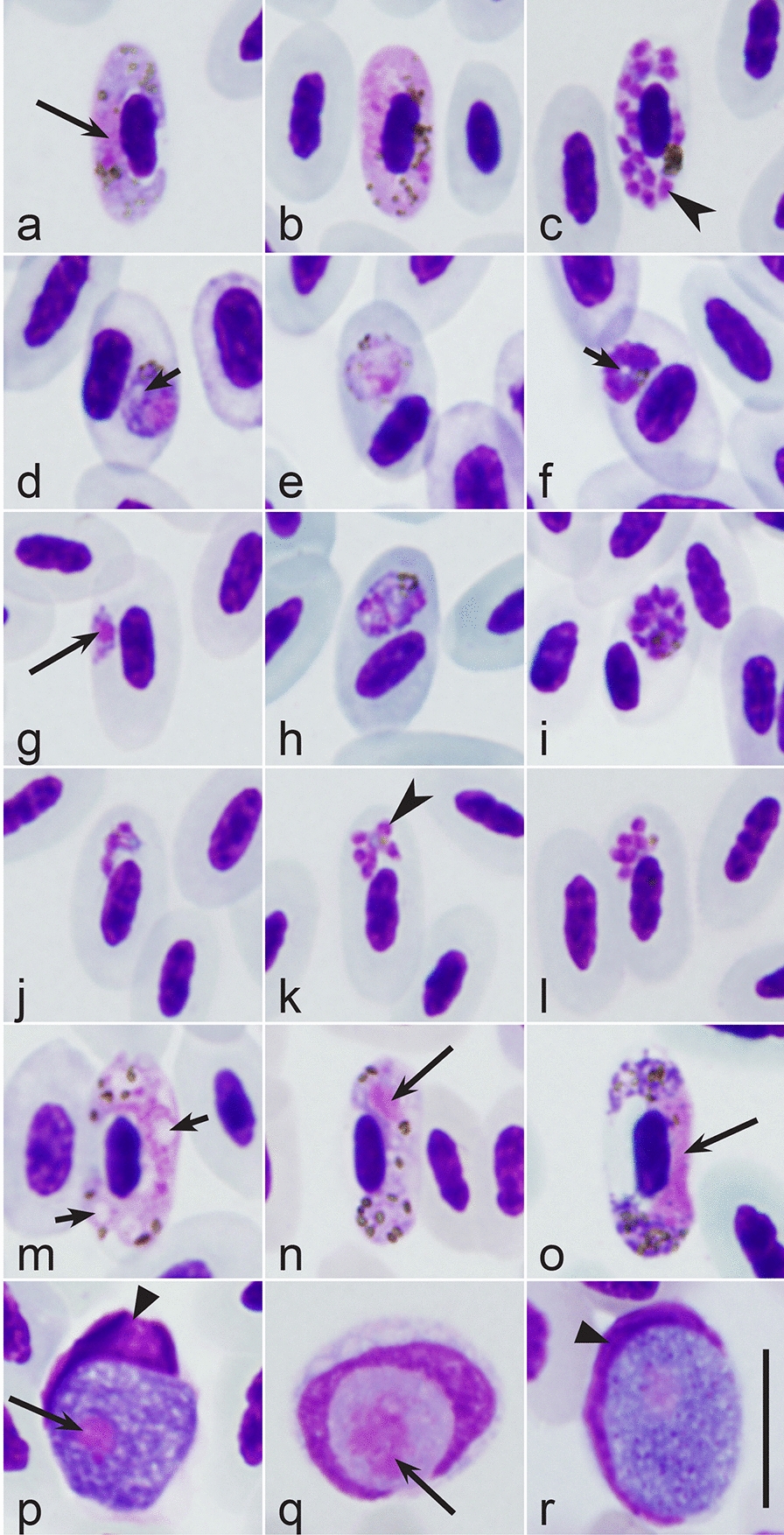
Gametocytes (**a, b, d, e, g, m–r**) and erythrocytic meronts (**c, f, h–l**) found in the blood films: *Plasmodium circumflexum* from Eurasian wren *Troglodytes troglodytes* (**a–c**), *Plasmodium matutinum* from European robin *Erithacus rubecula* (**d–f**), *Plasmodium relictum* (**g–i**) and *Plasmodium* (*Novyella*) sp. (**j–l**) from Coal tit *Periparus ater*, *Haemoproteus homopicae* from Eurasian jay *Garrulus glandarius* (**m**), *Haemoproteus majoris* from Great tit *Parus major* (**n**), *Haemoproteus attenuatus* from European robin (**o**). Unidentified to species *Leucocytozoon* parasites were seen in European robin (**p**), Eurasian jay (**q**) and Great tit (**r**). Note the circumnuclear shape of gametocytes and erythrocytic meronts in *P. circumflexum* (**a–c**); the presence of distinct vacuoles in gametocyte (**d**) and meront (**f**) of *P. matutinum*; the roundish shape of meront (**h, i**) of *P. relictum*, which markedly displace host cell nuclei; the tiny size of mature *P.* (*Novyella*) meronts (**j–l**); the marked vacuolization of the cytoplasm in gametocyte of *H. homopicae* (**m**); the broadly halteridial shape of mature *H. majoris* gametocyte (**n**); the attenuated shape of mature *H. attenuatus* gametocyte (**o**); the variously shaped host cell nuclei in *Leucocytozoon* parasites (**p–r**) infecting European robin (**p**), Eurasian jay (**q**) and Great tit (**r**). All images were from blood films of Group 2 birds. Long simple arrows—parasite nuclei, short simple arrows—vacuoles, simple arrowheads—merozoites, triangle arrowheads—host cell nuclei. Scale bar = 10 µm for all images

Parasites were identified according to keys [19, 24]. Representative blood films of the haemosporidians (Fig. 2, Table 2) were deposited in Nature Research Centre (accessions 49383–49393 NS).

**Table 2:** Diversity of avian malaria parasites (*Plasmodium* spp.) and related haemosporidians in two groups of passeriform birds migrating to wintering grounds from their breeding sites located between the White Sea and the Baltic Sea, northern Europe.

Bird family and species	Number of found infections		Difference *p*-value^a^	Parasite lineage found in birds of Group 2^b^
	Group 1, Curonian Spit, September 1979–1983	Group 2, Ventė Cape, September 2020		
Muscicapidae				
*Erithacus rubecula*	*Haemoproteus attenuatus* (1)	*Haemoproteus attenuatus* (1)	0.34 (0.5582)	hROBIN1
	*Plasmodium (Haemamoeba)* cf. *matutinum* (1) ^c^	*Plasmodium (Haemamoeba) matutinum* (1)	0.34 (0.5582)	pLINN1
	*Leucocytozoon* sp. (2)	*Leucocytozoon* sp. (1)	0.02 (0.8887)	lBT2
Paridae				
*Periparus ater* ^d^	*Haemoproteus* sp. (3)	*Haemoproteus* sp. (7)	2.23 (0.1354)	Not available ^e^
	*Plasmodium (Giovannolaia) circumflexum* (0)	*Plasmodium (Giovannolaia) circumflexum* (10)	**10.89 (0.0010)**	pTURDUS1
	*Plasmodium (Haemamoeba)* sp. (0)	*Plasmodium (Haemamoeba)* sp. (4)	3.48 (0.0621)	pGRW11
	*Plasmodium* sp. (0)	*Plasmodium* sp. (6) ^f^	**6.03 (0.0140)**	Not available
	*Plasmodium (Novyella)* sp. (0)	*Plasmodium (Novyella)* sp. (1)	0.04 (0.8474)	Not available
*Parus major* ^d^	*Haemoproteus majoris* (7)	*Haemoproteus majoris* (10)	0.12 (0.7296)	hPARUS1
	*Plasmodium (Giovannolaia) circumflexum* (0)	*Plasmodium (Giovannolaia) circumflexum* (15)	**10.70 (0.0011)**	pTURDUS1
	*Plasmodium (Haemamoeba)* sp. (2)	*Plasmodium (Haemamoeba)* sp. (1)	2.01 (0.1566)	Not available
	*Plasmodium* sp. (0)	*Plasmodium* sp. (2)	0.44 (0.5071)	Not available
	*Leucocytozoon* sp. (4)	*Leucocytozoon* sp. (2)	0.15 (0.6939)	lPARUS4
Troglodytidae				
*Troglodytes troglodytes* ^d^	*Plasmodium (Giovannolaia) circumflexum* (0)	*Plasmodium (Giovannolaia) circumflexum* (16)	**4.66 (0.0308)**	pTURDUS1
	*Plasmodium (Haemamoeba)* sp. (0)	*Plasmodium (Haemamoeba)* sp. (1)	0.21 (0.6466)	Not available
Corvidae				
*Garrulus glandarius*	*Haemoproteus* sp. (2)	*Haemoproteus* sp. (1)	0 (0.9543)	hGAGLA02
	*Haemoproteus homopicae* (0)	*Haemoproteus homopicae* (5)	2.96 (0.0853)	hGAGLA07
	*Plasmodium (Haemamoeba)* sp. (0)	*Plasmodium (Haemamoeba)* sp. (4)	2.11 (0.1462)	pSGS1
	*Leucocytozoon* sp. (11)	*Leucocytozoon* sp. (6)	0.54 (0.4630)	lCOCOR02

Data on number of found infections were obtained only by microscopic examination of blood films. Total number of examined bird species and additional information were given in Table 1

^a^Yates-corrected Chi-squire test value, followed in parenthesis by the corresponding significance *P*-value. Significant differences were shown in bold

^b^Results of PCR-based testing of best selected blood samples, which were microscopy positive

^c^Number of found infections during microscopic examination was given in parenthesis

^d^Number of *Plasmodium* infections in Group 2 exceeded the data given in Table 1 due to presence of co-infections in some bird individuals

^e^Presence of co-infections or unsuccessful amplifications precluded detection of some lineages by PCR-based methods

^f^Unidentified to subgeneric level parasites due to presence of only early blood stages. Some of them might belong to *Plasmodium (Giovannolaia) circumflexum*

The parasite lineage information was not available for birds of the Group 1 (samples of 1978–1983), because there were no PCR-based studies and blood samples were unavailable for DNA studies. The best-quality microscopically positive samples (parasitaemia ~ 0.01%, with parasite species or subgenus identified) collected in 2020 were examined using PCR-based methods for better understanding parasite genetic diversity in birds of the Group 2.

Standard nested PCR-based diagnostic protocols for detection of avian haemosporidian parasite lineages were used [43, 44]. Briefly, DNA was extracted from blood samples stored in SET-buffer using the ammonium-acetate protocol [45]. The primers HaemNR3/HaemNFI were used for the first PCR, while the primers HaemF/HaemR2 (for *Haemoproteus* sp. and *Plasmodium* sp.) and HaemFL/HaemR2L (for *Leucocytozoon* sp.) were used for the second PCR. 25 ng/μL of DNA was used as a template. All PCR profiles followed the original protocols. To control for PCR success and possible false amplifications, microscopically-positive *Haemoproteus* sp. and *Leucocytozoon* sp. samples (positive control) and ddH_2_O (negative control) were used. PCR products were run on a 2% agarose gel to determine the positive amplifications, which were then sequenced from 3ʹ and 5ʹ ends with a Big Dye Terminator V3.1 Cycle Sequencing Kit and ABI PRISM^™^ 3100 capillary sequencing robot (Applied Biosystems, Foster City, CA, USA). Sequences were checked for quality and possible presence of co-infections using software Geneious Prime 2020.0.5 (https://www.geneious.com). BLAST algorithms, which were available in MalAvi database (http://130.235.244.92/Malavi/) and Genbank (National Center for Biotechnology Information, National Institutes of Health, https://www.ncbi.nlm.nih.gov/genbank/) were applied to identify the lineages found. DNA sequences of parasite lineages found (Table 2) were deposited in GenBank (accessions OM311279-OM311295).

## Results

The overall prevalence of all haemosporidian infections of the genera *Haemoproteus, Plasmodium* and *Leucocytozoon* increased approximately 2.5-fold in birds of the Group 2 in comparison to the Group 1. However, no significant difference was discernible in the prevalence of *Haemoproteus* and *Leucocytozoon* when they were considered separately, but a 17.1-fold increase of the prevalence of *Plasmodium* infections was observed (Table 1). In other words, the overall prevalence of haemosporidians increased in the Group 2, entirely due to increase of the prevalence of malaria infections.

The prevalence of malaria infections only increased significantly in three bird species, i. e. the Coal tit*,* Great tit and Eurasian wren. A 24.5-fold increase of prevalence of malaria parasites was found in these three species in the Group 2 compared to the Group 1. No significant difference was discernible in prevalence of *Plasmodium* species in European robin and Eurasian jay (Table 1).

Due to light parasitaemia, parasite identification was possible mainly at genus and subgenus levels (Table 2). Similar gametocytes of *Haemoproteus* (Fig. 2m–o) and *Leucocytozoon* (Fig. 2p–r) species were seen in blood films of both bird groups (Fig. 2). Malaria parasites of subgenera *Haemamoeba* (Fig. 2d–i) and *Novyella* (Fig. 2j–l) were found, with no differences discernible in prevalence of these infections between the Groups 1 and 2. However, species of *Giovannolaia* (Fig. 2a–c) were not seen in birds of the Group 1, but were prevalent in birds of the Group 2 (Table 2). Even more, *P.* (*Giovannolaia*) *circumflexum* predominated and represented 55.5%, 100% and 94% of malaria-positive Coal tits, Great tits and Eurasian wrens, respectively. Neither this nor any other *Giovannolaia* species was seen in the same hosts 40 years ago. In other words, the increase of malaria infection in birds of the Group 2 is mainly due to *P.* (*G.*) *circumflexum,* which appears to be spreading.

The intensity of *Plasmodium, Haemoproteus* and *Leucocytozoon* species parasitaemia was low (< 0.001%) in most of the infections in both bird groups. This indicates a chronic stage of infections in all sampled birds. However, relatively high *P. circumflexum* parasitaemia ranging between 0.02% and 0.6% was seen in three individuals in the Group 2, but was not seen for any parasite in birds of the Group 1. This suggests presence of some active infections in birds of the Group 2.

Results of the PCR-based testing were mainly in accordance with the morphological identifications (Table 2, Fig. 2). The lineages of haemosporidians, which have been reported and often are common in Europe, were found, including *Plasmodium matutinum* (cytochrome *b* lineage pLINN1), *Plasmodium relictum* (pSGS1, pGRW11), *P. circumflexum* (pTURDUS1), *Haemoproteus attenuatus* (hROBIN1), *Haemoproteus homopicae* (hGAGLA07), *Haemoproteus majoris* (hPARUS1) as well as several *Haemoproteus* (hGAGLA02) and *Leucocytozoon* (lBT2) parasites that were not identified at species level. The lineages of several malaria parasites, seen in blood films (Table 2, Fig. 2), could not be identified due to the presence of co-infections, resulting in numerous double-base calling signals in sequence electropherograms, or unsuccessful amplifications (Table 2, Fig. 2).

## Discussion

This study reports the following key findings regarding changes of prevalence and diversity of avian haemosporidian parasites in northern Europe over the past 40-years: (i) the prevalence of *Haemoproteus* and *Leucocytozoon* parasites did not change significantly in any of the bird species studied; (ii) the prevalence of *Plasmodium* infections increased, but only in some bird species, (iii) the increase of malaria prevalence is entirely due to *P. circumflexum*. These findings are discussed below.

Despite 2.5-fold increase of the overall prevalence of haemosporidian parasites in the same bird species over the past 40-year period, the prevalence of *Haemoproteus* and *Leucocytozoon* infections did not change significantly (Table 1). This indicates a relatively stable epidemiological situation regarding haemoproteosis and leucocytozoonosis in this region of northern Europe. All the bird species examined are common and widespread in northern Europe and have been consistently ringed at the study sites ([38, 39, 42, 46], www.vros.lt, Ventės Ragas Ornithological Station database, accessed December 2021), so they are broadly available as hosts for *Haemoproteus* and *Leucocytozoon* infections. These bird species are flourishing in the Baltic Sea Region [40, 42, 46]. Similar prevalence of these parasites in both study groups (Table 1) suggests that the principal conditions, needed for their transmission, i. e. the availability of avian hosts, vectors, patterns of host-vector interactions and air temperature needed for sporogony have remained unchanged during the past 40 years. Haemosporidians of subgenera *Parahaemoproteus* (Fig. 2m–o) and *Leucocytozoon* (Fig. 2p–r) were present in both bird groups (Table 2). These parasites are transmitted by Ceratopogonidae biting midges (mainly by *Culicoides* spp.) and Simuliidae blackflies (mainly by *Simulium* spp.), respectively [19]. These blood-sucking insects are widespread in northern Europe [47, 48]. The unchanged prevalence of *Parahaemoproteus* and *Leucocytozoon* parasites in five species of birds suggests relatively similar availability of these vector groups.

It is interesting to note that both *Parahaemoproteus* and *Leucocytozoon* parasites can complete sporogony and produce infective stages (sporozoites) rapidly (within a week) at relatively low temperatures (about 15 °C and even less in some species of leucocytozoids), which are even optimal for some of these parasites [19, 49]. This feature of their biology suggests that a slight increase of air temperature probably should not influence these parasite transmission rate within the current level of climate warming, which has been recognized globally [29]. Climate in the Baltic Sea region also is changing [46, 50]. However, the main change signal currently is limited to slight temperature increase, particularly in spring and in the northern regions. It remains unclear if this might be considered a factor directly influencing haemosporidian parasite sporogony and their transmission. Humidity and precipitation levels did not change significantly in the studied area as well [46, 50], so hardly can be considered as the factors markedly affecting host birds and arthropod vectors ecology as well as epidemiology of haemosporidiosis. Other delicate factors related to vector-bird interactions might be more important but remain insufficiently studied*.* It is worth mentioning that transmission of *P. circumflexum* was not reported on the Curonian Spit 40 years ago [37], but occurs currently. A major ecological change at this study site is the maturation of forest, which consisted mainly of young pine-trees 40 years ago, but currently is a mature forest [19, 36–38]. It might be that mosquito vectors are becoming more successful in *P. circumflexum* transmission due to unknown factors, which emerge in mature forest ecosystems. However, this observation remains premature and needs further targeted research.

The overall prevalence of malaria parasites increased, but this was entirely due to a massive increase of the prevalence of *P. circumflexum* in three bird species, i.e. Coal tit*,* Great tit and Eurasian wren (Table 1). The prevalence of *P. circumflexum* or other *Plasmodium* parasites did not change significantly in the European robin and Eurasian jay. These findings indicate a notable change in the epidemiology of *P. circumflexum-*malaria in certain bird species, with no visible changes in other malaria infections. This finding is difficult to explain due to the insufficiently studied biology of *P. circumflexum.* The following observations are worthy of discussion.

It should be noted that the nests of the most prevalently infected three bird species (Coal tit*,* Great tit and Eurasian wren) usually are of relatively close-type; they often are located in various cavities, including tree-holes, rock-holes and also nest-boxes. Such nests are different from the more open-type nests of the European robin and Eurasian jay [51, 52]. There is only one report of *P. circumflexum* infection in the European robin [53], and this parasite has never been reported in the Eurasian jay. Ornithophilic mosquitoes bite birds and nestlings inhabiting various types of nests. They willingly enter nest-boxes, particularly often with nestlings [54]. Mosquito presence in the close nests might reduce the time needed for initiation of the exflagellation, ookinete development and sporogony of *P. circumflexum*, assuming that the insects remain in the nest following a blood meal for some time, particularly during the coldest period of a day (at night). Both the exflagellation and sporogony of malaria parasites is temperature dependent; their duration decreases in all *Plasmodium* spp. as temperature increases [1, 19, 55]. It is possible that the presence of parent-birds and nestlings contributes to the development of a microclimate with higher temperature conditions in the relatively small-space of the close-nests in comparison to open-air environments. Even a short-term temperature increase can be beneficial for successful initiation of the exflagellation and sporogony. Subsequent visits of infected mosquitoes to nests for blood meals might lead to the infection of whole nesthold with malaria parasites, including nestlings before they escape from the nests. This might be a factor contributing to spread of malaria parasites at high latitudes where outside daily temperature conditions are not optimal or restrictive for malaria transmission. This is not unexpected due to observations in regard of human malaria in countries with cold climates. For example, an endemic human *Plasmodium vivax* malaria existed as an indoor disease in Finland and northern Scandinavian countries in the nineteenth century [55]. The infected anopheline mosquitoes entered the peoples’ houses and survived there, resulting in successful sporogony during the cold season, and infection of the whole household. This observation calls for investigation of the behaviour of mosquito vectors and the success of sporogony in relation to the nesting behaviour of birds. This study suggests that *P. circumflexum* (lineage pTURDUS1) could be a model organism for such research.

The vectors of the *P. circumflexum* lineage pTURDUS1 are unknown, but observations have shown that unidentified lineages of this species can complete sporogony and produce sporozoites in *Culiseta* and *Mansonia* mosquitoes [56], so these might be responsible for transmission of lineage pTURDUS1. However, there is still no proof of the involvement of *Culiseta* and *Mansonia* species in malaria transmission in northern Europe. *Culiseta* mosquitoes are present and abundant at breeding areas of the studied bird species [57].

It is worth noting that *P. circumflexum* (lineage pSW5) is present in birds breeding in Northern Japan and probably is transmitted by *Culiseta* species [58]. Moreover, *P. circumflexum* (lineage pBT7) has been recently found in *Culiseta* sp. in Alaska, showing that these mosquitoes took infected bird blood meal [59]. The lineage pBT7 is closely related to the lineage pTURDUS1. These lineages differ only by 1 base pair in the 478 bp of the cytochrome *b* bar-coding region, and both parasites produce erythrocytic meronts of similar morphology, hence likely represent different strains of the same pathogen species. The reports of *P. circumflexum* in wild-caught *Culiseta* sp. correspond to the former experimental observations about vectorial ability of *Culiseta* mosquitoes to transmit this pathogen [56], suggesting that *Culiseta* spp. might be important vectors in Alaska and probably other countries with cold climates. Numerous malaria PCR-positive signals were found in tested *Culiseta* spp., including a report from one head/thorax sample, indicating possible presence of sporozoites, which occur in salivary glands of mosquitoes [59]. Importantly, the presence of blood stages of *P. circumflexum* was documented in Black-capped chickadee (*Poecile atricapillus*), a tree-hole breeder belonging to the Paridae [59]. These data coincided with high prevalence of *P. circumflexum* in the two hole-breeder species of the Paridae in our study (Table 1). Loiseau et al. [32] found *P. circumflexum* (lineage pBT7) in Alaskan resident birds above the Arctic Circle, suggesting that this malaria parasite species is cold-tolerant and capable of northward distribution. The lineages pBT7 and pTURDUS1 were common in Blue tits *Cyanistes caeruleus,* which, like the Great tit and Coal tit, breed in nest boxes [20]. The available published information and this study (Tables 1, 2) call for further field and experimental research on vector biology of avian malaria parasites, which are transmitted and expand areas of transmission at high latitudes. *Culiseta* species are cold-adapted, often ornithophilic [60, 61] and support sporogony of *P. circumflexum* [56, 62], but remain insufficiently investigated as vectors of avian malaria at high latitudes.

It is difficult to speculate whether and how the reported increase of the *P. circumflexum* (pTURDUS1) prevalence might be related with health of birds examined during this study. Limited experimental observations showed that virulence of different strains and isolates of *P. circumflexum* varied remarkably in different avian hosts, ranging from invisible symptoms to severe disease and even mortality [19, 63, 64]. The lineage pTURDUS1 caused high parasitaemia in experimentally infected Eurasian siskins *Carduelis spinus* (maximum parasitaemia of 30% was reported) and the Common crossbills *Loxia curvirostra*, but low transit parasitaemia developed in domestic ducklings *Anas platyrhynchos* (0.01%) [65] and domestic canaries (G. Valkiūnas, pers. ob.). Further experimental and field studies are needed for better understanding the biology of this common generalist malaria pathogen, which has been reported in 44 bird species in MalAvi database (http://130.235.244.92/Malavi, accessed in December 2021). In northern Europe (55 °N and above), pTURDUS1 has been found in Norway, Sweden, Russia, Finland and Lithuania according to the MalAvi database.

It is important to note that *P. circumflexum* has been found in many bird species in central and southern Europe but has never been reported in Europe north of 55°N in the past century [66, 67]. The present study shows that prevalence of this infection increased remarkably in northern Europe during the past 40 years. Moreover, the reports of this parasite in hatched year birds certainly revealed the local transmission in northern Europe. *Plasmodium circumflexum* (pTURDUS1) was recently found in European robin, Great tit and Eurasian wren in Sweden [53]. This parasite was found for the first time and molecularly characterized due to isolation from Eurasian wren sampled on the Curonian Spit in 2007 [68]. It has never been found in Eurasian jay, indicating a possible resistance or ecological isolation of this avian host from vectors. This study suggests that *P. circumflexum* (pTURDUS1) is spreading and should be considered as an invasive bird malaria pathogen in northern Europe. The same process probably occurs in North America in regard of the closely related lineage pBT7 [32, 59]. An unknown lineage of *P. circumflexum* was reported in America in the past century, but not north of 55°N, as also was the case in Europe [62, 66, 67, 69–71].

Malaria parasites of the subgenus *Haemamoeba* (Fig. 2d–i) were found in both bird groups, with no significant changes in their prevalence (Table 1). Only one *Novyella* infection (Fig. 2j–l) was seen (Table 2). These data indicate a relatively similar rate of transmission of these parasites in studied birds during the past 40 years.

This study was based on samples collected during autumn (September) when transmission of avian haemosporidians slow down or even might interrupt due to the decrease of activity of the vectors [19] and terminated bird breeding [40] at high latitudes. Parasitaemia was low in all reported haemosporidian infections, which were likely turning to a chronic stage. However, several active *P. circumflexum* infections (parasitaemia between 0.02% and 0.6% or 2 and 60 parasites per 10,000 red blood cells) were still reported in the second bird group, but were absent in the first group. This preliminary observation might indicate some prolonged period of *P. circumflexum-*malaria transmission, resulting in observation of relatively high parasitaemia during autumnal bird migration. Further field studies are needed for better understanding if time of transmission is extending in the wild.

This study (Table 2, Fig. 2) further shows need for combining methods of microscopic examination and molecular detection in ecology research of haemosporidian parasites [43]. For example, *Novyella* species was readily visible in blood films (Fig. 2j–l), but the parasite lineage was not detected (Table 2) due to co-infection with *P. circumflexum* (pTURDUS1). Application of both tools in parallel increases opportunities of parasite diagnostics, particularly during co-infections, which are common in the wild and even predominate in some bird populations [6].

At high latitudes, the field work aiming for collection of samples for avian malaria research has been non-popular in autumn due to low prevalence of *Plasmodium* spp. infections. Most studies focused on the malaria parasite sampling during a spring–summer period when haemosporidian infections are more prevalent and parasitaemia is high [19, 72]. The present study shows that (i) prevalence of *P. circumflexum* increased during the past 40 years and (ii) the malaria infected birds are common and can be readily sampled in autumn. This opens opportunities to extend a period of fieldwork aiming, for example, at the search for malaria parasites donors (infected bird individuals), which are necessary for parasite strains isolation and other avian malariology issues. Avian malaria field studies are worth planning not only during spring–summer period in northern Europe, as was a common case before, but also during early autumn (September) when bird populations increase due to the presence of juveniles and bigger number of bird individuals can be accessed for research during a short period of time.

## Conclusion

Similar prevalence and diversity of *Haemoproteus* and *Leucocytozoon* infections were reported in the same bird species and ages sampled at the same area and season 40 years apart. This suggests existence of a relatively stable epidemiological situation of these infections in northern Europe. The same data were obtained for malaria parasites of genus *Plasmodium* belonging to *Haemamoeba* and *Novyella* subgenera, except for one species—*Plasmodium* (*Giovannolaia*) *circumflexum*, whose prevalence increased remarkably during this period of time. *Plasmodium circumflexum* infection is spreading selectively in regard of different avian hosts, being prevalent in Coal tit, Great tit and Eurasian wren, but rare or absent in European robin and Eurasian jay. *Plasmodium circumflexum* seems to be an invasive avian malaria pathogen in northern Europe, but its transmission biology and mosquito vectors remain insufficiently understood. The high prevalence of *P. circumflexum* in birds breeding in relatively close-nests suggests a possible important role of the nesting biology in this pathogen transmission. This study calls for extending of research aiming better understanding of epidemiological situation on *P. circumflexum-*malaria at high latitudes.

## Data Availability

All data generated during this study are included in this published article and can be available on request.
